# Fucoidan Extracted from the New Zealand *Undaria pinnatifida*—Physicochemical Comparison against Five Other Fucoidans: Unique Low Molecular Weight Fraction Bioactivity in Breast Cancer Cell Lines

**DOI:** 10.3390/md16120461

**Published:** 2018-11-22

**Authors:** Jun Lu, Keyu Kally Shi, Shuping Chen, Junqiao Wang, Amira Hassouna, Loretta Nicole White, Fabrice Merien, Mingyong Xie, Qingjun Kong, Jinyao Li, Tianlei Ying, William Lindsey White, Shaoping Nie

**Affiliations:** 1 College of Life Sciences and Oceanography, Shenzhen University, Shenzhen 518071, China; 2 School of Science, Faculty of Health and Environmental Sciences, Auckland University of Technology, Auckland 1010, New Zealand; ffx0876@autuni.ac.nz (K.K.S.); loretta.white@aut.ac.nz (L.N.W.); fabrice.merien@aut.ac.nz (F.M.); kongqingjun1976@snnu.edu.cn (Q.K.); lindsey.white@aut.ac.nz (W.L.W.); 3 College of Food Engineering and Nutritional Science, Shaanxi Normal University, Xi’an 710119, China; 4 School of Interprofessional Health Studies, Faculty of Health and Environmental Sciences, Auckland University of Technology, Auckland 1010, New Zealand; amira.hassouna@aut.ac.nz; 5 Institute of Biomedical Technology, Auckland University of Technology, Auckland 1010, New Zealand; 6 State Key Laboratory of Food Science and Technology, Nanchang University, Nanchang 330047, China; ncuchenshuping@sina.com (S.C.); ncuskwangjunqiao@163.com (J.W.); myxie@ncu.edu.cn (M.X.); 7 Department of Medical Biochemistry and Molecular Biology, Faculty of Medicine, Cairo University, Cairo 12613, Egypt; 8 AUT-Roche Diagnostics Laboratory, School of Science, Faculty of Health and Environmental Sciences, Auckland University of Technology, Auckland 1010, New Zealand; 9 Xinjiang Key Laboratory of Biological Resources and Genetic Engineering, College of Life Science and Technology, Xinjiang University, Urumqi 830046, Xinjiang, China; ljyxju@xju.edu.cn; 10 Key Laboratory of Medical Molecular Virology of MOE/MOH, Shanghai Medical College, Fudan University, 130 Dong An Road, Shanghai 200032, China; tlying@fudan.edu.cn

**Keywords:** fucoidan, chemical composition, molecular weight, breast cancer cell lines, nutraceutical

## Abstract

Fucoidan, the complex fucose-containing sulphated polysaccharide varies considerably in structure, composition, and bioactivity, depending on the source, species, seasonality, and extraction method. In this study, we examined five fucoidans extracted from the same seaweed species *Undaria pinnatifida* but from different geological locations, and compared them to the laboratory-grade fucoidan from Sigma (S). The five products differed in molecular composition. The amount of over 2 kDa low molecular weight fraction (LMWF) of the New Zealand crude fucoidan (S1) was larger than that of S, and this fraction was unique, compared to the other four fucoidans. The difference of molecular compositions between S and S1 explained our previous observation that S1 exhibited different anticancer profile in some cancer cell lines, compared with S. Since we observed this unique LMWF, we compared the cytotoxic effects of a LMWF and a high molecular weight fucoidan (HMWF) in two breast cancer cell lines—MCF-7 and MDA-MB-231. Results indicated that the molecular weight is a critical factor in determining the anti-cancer potential of fucoidan, from the New Zealand *U. pinnatifida*, as the LMWF exhibited a dose-dependent inhibition on the proliferation of breast cancer cells, significantly better than the HMWF, in both cell lines. A time-dependent inhibition was only observed in the MCF-7. Induction of caspase-dependent apoptosis was observed in the MDA-MB-231 cells, through the intrinsic apoptosis pathway alone, or with the extrinsic pathway. LMWF stimulated a dose-dependent NOS activation in the MDA-MB-231 cells. In conclusion, the fucoidan extracted from the New Zealand *U. pinnatifida* contains a unique LMWF, which could effectively inhibit the growth of breast cancer cell lines. Therefore, the LMWF from New Zealand *U. pinnatifida* could be used as a supplement cancer treatment.

## 1. Introduction

Fucoidans are complex series of fucose-containing sulphated polysaccharides, mainly found in the cell wall [1] and intercellular spaces of brown seaweeds [2] and are the main components of the sticky substance in brown seaweeds [1]. Here, it can make up to over 40% dry weight of the seaweed cell walls [3] and up to 16% dry weight of the whole alga [4]. It is also found in marine invertebrates like sea cucumbers (body wall) [5]. One of the common sources to extract fucoidan that is used to manufacture the fucoidan nutraceutical products on the market, is the seaweed species *Undaria pinnatifida.* This seaweed is farmed extensively in Asia and generates in excess of US$1.6 billion value per annum, primarily as a food (Wakame) [6]. *U. pinnatifida* was introduced to New Zealand in the 1980s and has, since, spread throughout the country. It is classed as an unwanted organism under the Biosecurity Act 1993, section 164c [4]. Since 2010, it has been permitted to be harvested from artificial structures e.g., aquaculture farms, and with farming permitted in heavily-infested areas [7]. This has led to a growing interest in the production of fucoidan from the New Zealand *U. pinnatifida* and a pilot-scale commercial production of fucoidan, from the seaweed [8].

Fucoidan has numerous proven bioactivities, such as antioxidant [9], anticoagulant [10], antiviral [11] and anticancer [12] activities. These bioactivities are linked to the molecular weight (MW) [13], composition (e.g., monosaccharide composition, the degree of sulphation) [14], and structure (glycosidic linkages, the degree of branching and substitution, chain conformation, etc.) [15]. However, it is known that the fucoidan varies significantly between the source species, on each of these three parameters—the environment, the source seaweeds from where they were cultivated or harvested, and even the time of the year [16]. No two isolated fucoidans are exactly the same, even if they are extracted from the same seaweed species; they are all unique in their structure, composition, and bioactivities [17].

We conducted a previous study that showed that fucoidan extracted from New Zealand *U. pinnatifida* has different in vitro anticancer profile, compared with the fucoidan supplied from Sigma, which was also extracted from *U. pinnatifida*, but grown elsewhere [18]. The study assumed that lower the MW of the purified fucoidan fraction, higher the cell reducing capacity. Several studies [19,20,21] have indicated that the major role of sulphate groups in cancer cell suppression, and the large MW fucoidan polymer, might hide the anionic sulphate groups inside its spherical conformation, hindering the reaction of the sulphate groups with the cancer cells. On the other hand, the LMWF are more likely to exist in a loose and linear form [22]. Additionally, the LMWFs were found to be more water soluble than the HMWFs, which affects their bioavailability [23,24]. Thus, the production of lower-sized oligosaccharides is an important factor for the development of more feasible anti-cancerous agents.

Anti-cancer properties of fucoidan in breast cancer have been reported in both in vitro and in vivo studies, involving various cell signalling pathways. Fucoidan isolated from the *Cladosiphon okamuranus* inhibited the proliferation of the MCF-7 cells, in a time- and dose-dependent manner, and induced apoptosis, through the extrinsic pathway. Meanwhile, it showed no cytotoxic effect on normal human mammary epithelial cells [25]. Fucoidans from *Saccharina japonica* and *U. pinnatifida* (derived from East Asia) inhibited both cell proliferation and colony formation in the T-47D breast cancer cells. Along with its cytotoxic effects, fucoidan was proven to block the MDA-MB-231 breast carcinoma cells’ adhesion to platelets, which implied its potential for tumour metastasis suppression [26]. In animal models, fucoidan extracted from the *Fucus vesiculosus* inhibited the 4T1 mouse breast cancer cell growth, in vivo and in vitro, via the downregulation of the Wnt/β-catenin signalling pathway, without causing cytotoxic effects in normal cells. A decrease of the vascular endothelial growth factor (VEGF) expression was also observed in the 4T1 cells, indicating the antiangiogenic activity of the fucoidan [27].

As a non-toxic anti-cancer agent, fucoidan can be used in combination with chemotherapy agents (including endocrine/targeted therapies) to lower the toxicity of therapy to patients, as well as generate synergistic inhibitory effects on breast cancer. A recent study has reported a combination treatment of fucoidan (obtained from Japan) and three chemotherapeutic agents (cisplatin, tamoxifen, and paclitaxel) on two breast cancer cell lines (MCF-7 and MDA-MB-231). Compared to the use of treatments with fucoidan or drugs alone, this combination treatment exhibited highly synergistic inhibitory effects on the growth of breast cancer cells. It has been stated that fucoidan enhances the downregulation of the anti-apoptotic proteins Bcl-xL and Mcl-1, through the use of these chemotherapeutic drugs and the intracellular ROS levels, and reduced glutathione (GSH) levels in breast cancer cells. A protective effect of the normal human fibroblast TIG-1 cells, by fucoidan, to prevent apoptosis from cisplatin and tamoxifen has also been observed, indicating a decrease in the side effects of therapy [23]. The anti-metastatic property of fucoidan is also a promising quality to improve the overall survival for patients, especially for the metastatic breast cancer (MBC) patients. Taken together, these outcomes suggest a favourable characteristic of fucoidan, for its application in breast cancer treatment.

The majority of studies have failed to characterize the chemical structure of the fucoidan under study, due to its branched and heterogeneous nature. Therefore, drawing a conclusion about the relation between the structural characteristics and the specific bioactivities of the isolated fucoidans, have encountered great difficulties [28]. It is also difficult to compare the fucoidan activity of crude and purified forms, therefore, it is necessary to investigate the structure, composition, and bioactivity of the purified fucoidan [29]. Moreover, results from many studies suggest that the bioactivity of fucoidan on cancer cells, depends on the fucoidan source, and it is cell-type specific [28,30,31,32,33]. Hence, the ongoing research to characterize the fucoidan chemical structure and to discover the possible mechanisms by which the LMW fucoidan polymers result in cytotoxicity in different tumour cells, as a fucoidan with lower potential contaminants and a higher antitumour activity will be a safe and suitable material, for medical applications of fucoidan, in cancer patients. Therefore, in this study, we aimed at comparing the physicochemical and molecular weight compositions of fucoidan produced from the New Zealand *U. pinnatifida*, with five commercially-produced fucoidan extracted from the same species but grown in different regions of the world. Based on the comparison, we then further studied the cytotoxic effects of the LMWF and the HMWF of the New Zealand *U. pinnatifida*, on two breast cancer cell lines, as information is sparse in this area. We also tried to identify the possible cytotoxicity mechanisms in one of the two breast cancer cell lines, MDA-MB-231, using molecular biology tools, such as a flow cytometer. 

## 2. Results and Discussion

### 2.1. Chemical composition

The chemical composition of all samples is summarised in Table 1. The fucoidan samples consisted mostly of carbohydrates (44.7–96.8%), sulphates (15.5–25.6%) with minor amounts of uronic acid (1.6–4.2%), protein (trace to 3%), and fat (0–2.3%), with the exception of S2, which was the fucoidan sample purchased through the Alibaba website from a Chinese manufacturer, which claimed to be of 75.5% purity. Contrary to the claim, sulphate, one of the key characteristics of fucoidan, was absent. In terms of the monosaccharide composition, the most abundant monosaccharides in all samples, were fucose and galactose, except for S2, which consisted mainly of glucose (96.7%). Given that in any fucoidan, fucose is the main monosaccharide, we concluded that S2 could not be categorised as fucoidan.

There was a clear pattern that the ratio of fucose and galactose was close to 1:1, in all samples in which they were present. This agreed with previous findings in the same species [34,35]. The fucose content of the New Zealand fucoidan produced by us (S1, 19.5%) was the second highest of all samples, in comparison to the Sigma reference sample (27%), and very close to the highest (S4, 20.4%), which was produced by Marinova.

The sulphate content of the fucoidan produced by us (S1, 19.7%) was also compatible with the Marinova sample (21%), as well as the Sigma sample (26%). The sulphate content of the *U. pinnatifida* has been recorded to be from 9.18% [35], 10.4% [34], 25% [27], 34.6% [9], to 41.5% [36]. The sulphate content of S3, a fucoidan produced by a local New Zealand business, Matakana SuperFoods, had the lowest sulphate content of all samples (15.5%), apart from S2. However, it was still within the known-range. Sulphation level was regarded to be positively correlated to the fucoidan’s bioactivity [17]. Hence, of all the samples analyzed, S2 should not have contained any fucoidan-related bioactivity, while others were likely to possess certain degrees of the fucoidan-related bioactivity.

In terms of the total uronic acid content, S1 (1.8%) was the closest to the Sigma reference sample (1.6%). S3 (Matakana fucoidan) and S4 (Marinova fucoidan) had a similar amount of uronic acid, they were also the only two samples that contained glucuronic acid in the form of uronic acid. Our fucoidan product S1, had a trace amount of glucuronic acid, which was different from our previous laboratory-scale extraction results, where the uronic acid content of the fucoidan was compared by different methods, using glucuronic acid as a standard. The results varied between 2.1% to 4.9% [9,18].

Table 2 shows the chemical composition of the HMWF, LMWF, and the fucoidan having a MW between 10–300 kDa. The LMWF had a lower sulphate content than the HMWF, however, it was more likely to exist in a loose-conformation, allowing the sulphate groups to interact with the cancer cells [22].

### 2.2. Analysis of the Elements and Organic Pollutants

Across all six samples, the most abundant elements were the essential minerals for human health, such as Ca, K, Na, and Mg, while the content of heavy metals Cr, Pb, Cd, Hg, and As, ranged from low to trace amounts (Table 3). The Sigma sample had the most desirable profile as expected, with the maximum levels of sulphur, and the least amount of heavy metals and organic pollutants (trace amounts to not detected at all). The K level of the Sigma sample was significantly lower than the rest of the samples, on the contrary, the Na level of the Sigma sample was significantly higher than that of the other samples. Our fucoidan product sample (S1) had the third highest amount of sulphur (~84 ppm), following the Sigma reference sample (S, ~103 ppm), and the Marinova sample (S4, ~87 ppm); it also had the second highest amount of K (~54 ppm); the highest level of Ca (~47 ppm), and the lowest amount of Mg (~1 ppm). In terms of the heavy metals, S1 was comparable with the Marinova sample, with significantly lower levels of Cd, Hg, and As; and relatively lower levels of Pb and Cr.

In addition to not qualifying as a “fucoidan”, S2 (purchased through the Alibaba website from a Chinese manufacturer) had the highest amount of Pb, which exceeded the safety limits of Chinese seafood standard (10 mg/kg), and the recommendation of the tolerable weekly intake (based on 70 kg body weight) by World Health Organisation (WHO) and the Food and Agriculture Organisation of the United Nations (FAO).

### 2.3. Molecular Weight

**Figure 1:**
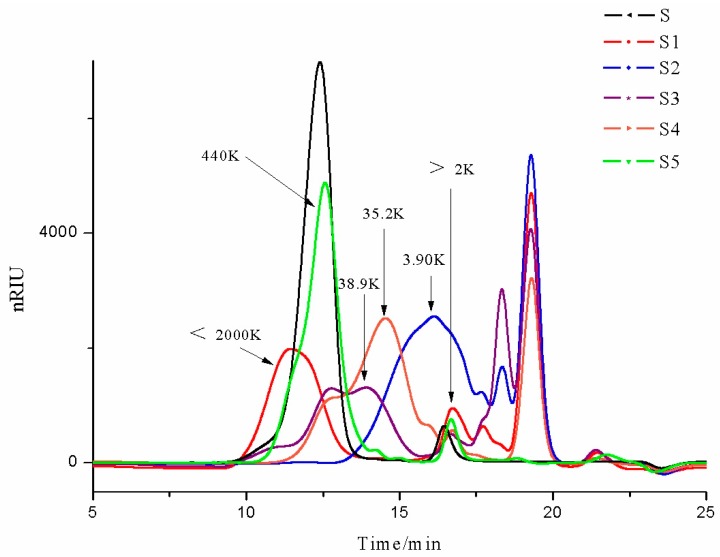
Profiles of the six fucoidans, monitored by high-performance gel permeation chromatography (HPGPC), coupled with a refractive index detector (RID). S = *Undaria pinnatifida* fucoidan (≥95%, CAS: 9072-19-9) purchased from Sigma Aldrich; S1 = crude fucoidan from our pilot industrial-scale production in New Zealand; S2 = fucoidan purchased through the Alibaba website from a Chinese manufacturer; S3 = fucoidan produced by a local New Zealand business, Matakana SuperFoods; S4 = fucoidan (GF100, minimal purity 75%) produced by Marinova Ltd. (Cambridge, Tasmania, Australia); S5 = fucoidan purchased from Leili Ltd. (Beijing, China).

**Figure 2:**
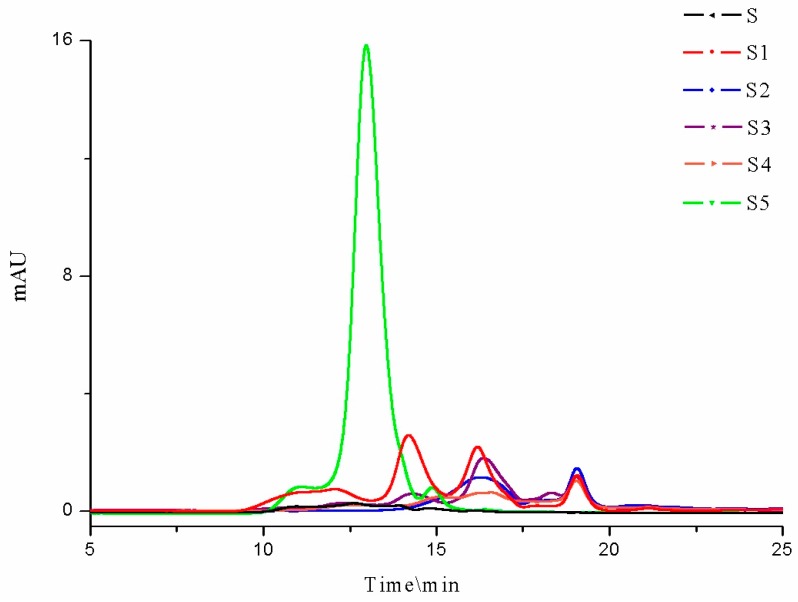
The HPGPC profiles of the six fucoidans monitored by a UV detector. S = Undaria pinnatifida fucoidan (≥95%, CAS: 9072-19-9) purchased from Sigma Aldrich; S1 = crude fucoidan from our pilot industrial-scale production in New Zealand; S2 = fucoidan purchased through the Alibaba website from a Chinese manufacturer; S3 = fucoidan produced by a local New Zealand business, Matakana SuperFoods; S4 = fucoidan (GF100, minimal purity 75%) produced by Marinova Ltd. (Cambridge, Tasmania, Australia); S5 = fucoidan purchased from Leili Ltd. (Beijing, China).

The molecular weight (MW) of fucoidan is another important parameter that influences bioactivity. High-performance gel permeation chromatography (HPGPC) results (Figure 1 and Figure 2) indicated that all fucoidan samples consisted of, both, a relatively higher MW fraction and a lower MW fraction (>2 kDa). The Sigma reference sample (S) and the fucoidan purchased from Leili Ltd. (S5, Beijing, China) exhibited similar elution profiles, with a major large MW fraction around 440 kDa, a minor small MW fraction over 2 kDa, and both showed a lack of MW fractions lower than 2 kDa. S1 also contained a major large MW fraction with MW between 440 and 2000 kDa. The amount of over 2 kDa minor small MW fraction of S1 appeared to be larger than that of S and S5. S1 also had small molecules that were under 2 kDa. It has been suggested that low MW fractions of fucoidan had a more potent bioactivity compared to the high MW fractions of fucoidan. Hence, the difference of the molecular composition between S and S1, with the presence of this unique LMW fraction in S1, may explain our previous observation that our fucoidan exhibited different anticancer profiles, in some cancer cell lines, compared to Sigma fucoidan [18]. The Matakana fucoidan (S3) contained a high MW fraction around 440 kDa, but also contained a more prominent fraction with a MW around 38.9 kDa. It had two small MW fractions, one being a small fraction over 2 kDa, and the other being a relatively large fraction under 2 kDa. Marinova fucoidan (S4) exhibited a similar pattern in the large MW fraction, with a smaller peak at 440 kDa and a larger peak at 35.2 kDa. It contained a small over 2 kDa low MW fraction peak, but a lack of under 2 kDa fraction, which was similar to S and S5.

S5 had the strongest UV absorption (Figure 2), indicating the highest protein content, which was in accordance with the results of the BCA assay (Table 1). The protein in this fucoidan might be conjugated with the high MW fraction. The other fucoidan samples also exhibited absorption that indicated the presence of a low level of protein. This might be linked to a difference in the extraction methods and also the time and location of the seaweed harvest, as we have shown previously that there is a seasonal change in the protein content of the *U. pinnatifida* [37].

### 2.4. Molecular Weight Related Bioactivity of Fucoidans

**Figure 3:**
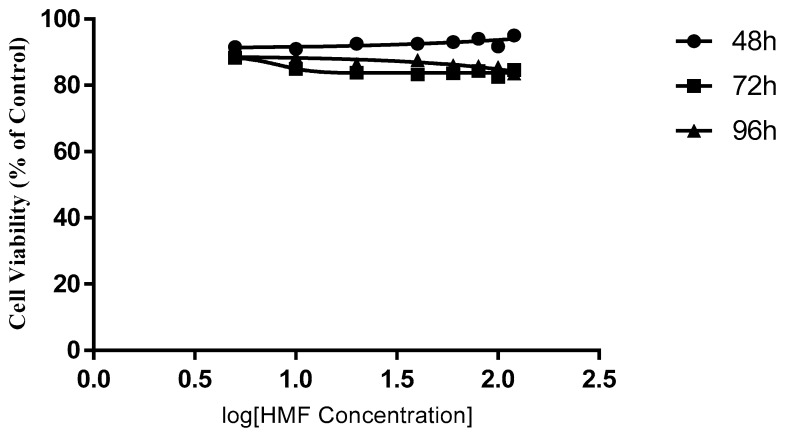
The effect of high molecular weight fraction (HMWF) on the MCF-7 cell line. Data are presented as means ± S.D., n = 6.

**Figure 4:**
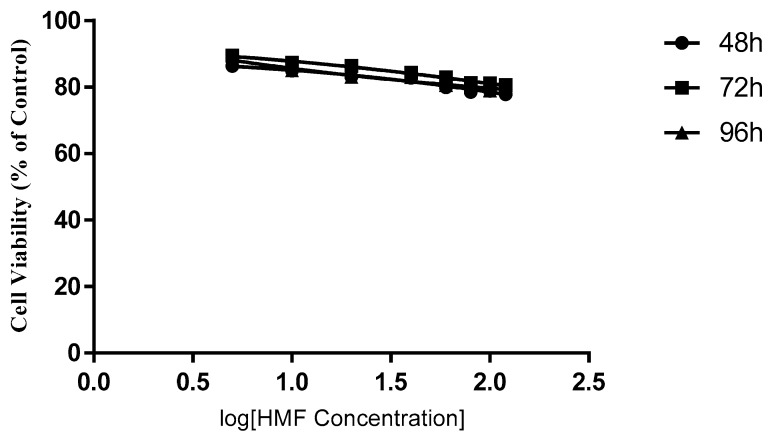
The effect of HMWF on the MDA-MB-231 cell line. Data are presented as means ± S.D., n = 6.

The cytotoxic effects of HMWF and LMWF were significantly different in, both, the MCF-7 and the MDA-MB-231 cell lines. In both cell lines HMWF had a very limited effect on slowing down the growth of cancer cells (Figure 3 and Figure 4). In the MCF-7, no obvious dose-dependent inhibition was observed after an incubation of 48 and 72 h (p = 0.1818 & p= 0.2738, One-way ANOVA). Cell viabilities had only dropped by less than 8.5%, after being treated with HMWF in various concentrations, for 48 h and 17.5% for 72 h. After 96 h, the cell viability had decreased in a slightly wider range from 5 µg/mL to 120 µg/mL groups (p = 0.01281, one-way ANOVA). The results indicated that, in general, HMWF did not cause much difference on the inhibition of cells treated with various concentrations (PCn = 0.1868). In the MDA-MB-231, the inhibitory effect of the HMWF cells was similar to the inhibition on the MCF-7 cells. A slight dose-dependent decrease (86.39% to 77.91%, with HMWF from 5 µg/mL to 120 µg/mL) was observed, after incubation of 48 h, and remained nearly unchanged up to 72 h, and until 96 h. However, the most significant inhibition with the HMWF treatment was still around 20%, far from enough to become a potential therapeutic agent for cancer treatment.

In the MCF-7 cell line, cells were treated with LMWF, from a range of the highest 200 µg/mL to the lowest 0.5 µg/mL. The inhibitory effect was most significant at the LMWF concentrations of 200 µg/mL, after incubation of 96 h (cell viability dropped to 42.42%, compared to the negative controls) and was at a very close value after 72 h (43.38%). The IC_50_ after 96 and 72 h, was 10.540 µg/mL and 19.087 µg/mL of the LMWF, respectively. The best inhibition rate at 48 h was 55.23%, at 200 µg/mL of LMWF, therefore, the IC_50_ for cells incubated for 48 h would be over 200 µg/mL. The MCF-7 showed a good sensitivity to the LMWF, with 0.5% µg/mL LMWF inhibiting the cell growth by 18%, at 72 h and 22% by 96 h.

**Figure 5:**
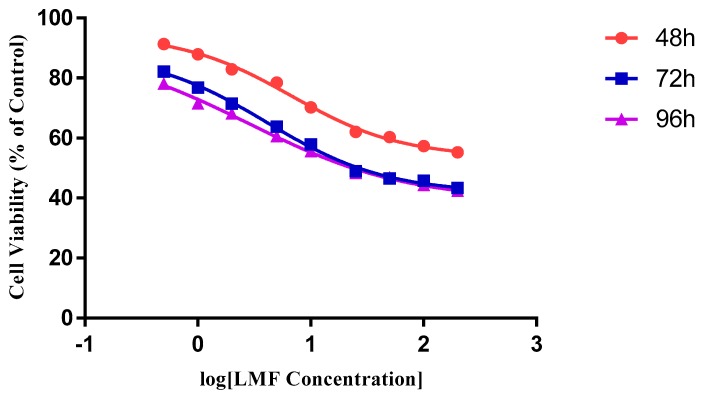
The effect of low molecular weight fraction (LMWF) on the MCF-7 cell line. Data are presented as means ± S.D., n = 6.

**Figure 6:**
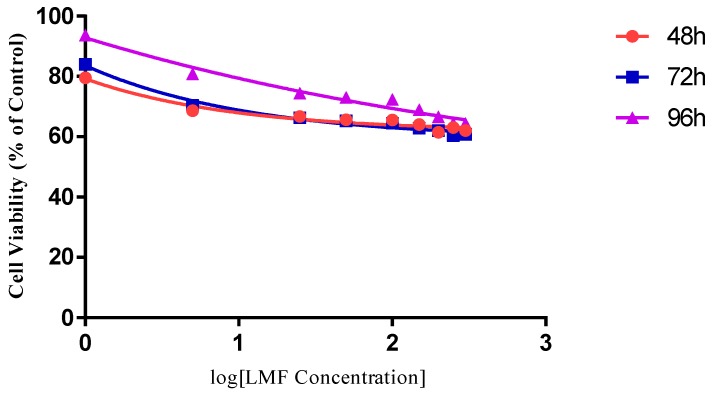
The effect of LMWF on the MDA-MB-231 cell line. Data are presented as means ± S.D., n = 6.

The LMWF exhibited a great suppression on the cell viability, in both cell lines (Figure 5 and Figure 6). The dose-dependent inhibitions of cell proliferation have been observed in each time point of 48, 72, and 96 h, in both types of cells. In the MCF-7 group, a decrease of the cell population has been observed in the cells treated with all LMWF concentrations (from 200 µg/mL to 0.5 µg/mL) from 48 h to 72 h. The inhibitory effect was most significant at the LMWF concentration of 200 µg/mL after incubation of 96 h (cell viability dropped to 42.42%, compared to the negative controls) and was at a very close value after 72 h (43.38%). The IC_50_ after 96 and 72 h was 10.540 µg/mL and 19.087 µg/mL of the LMWF, respectively. The best inhibition rate at 48 h, was 55.23% at 200 µg/mL of the LMWF, therefore, the IC_50_ of the cells incubated for 48 h would be over 200 µg/mL. The MCF-7 showed a good sensitivity to the LMWF, with 0.5% µg/mL LMWF inhibiting the cell growth by 18%, at 72 h and 22% by 96 h. Thus, these data show that the effect of LMWF concentrations on the inhibition in MCF-7 cells, was significant (PCn = 0.0283) and was in a very significant time-dependent manner (PTime < 0.0001), with no interaction of the effect of LMWF concentrations and incubation time (PTime:Cn = 0.3624).

The MDA-MB-231 cells were treated with LMWF from a range of highest 300 µg/mL to lowest 1 µg/mL. The inhibitory effect of LMWF was most significant at a LMWF concentration of 300 µg/mL, after incubation of 72 h (cell viability dropped to 60.76%, compared to the negative controls), although the inhibition rates at 48 and 96 h were very close to this value (62.01% and 64.41%, respectively). The IC_50_ of LMWF for the MDA-MB-231 cells would be above 300 µg/mL, for all incubation times. Although the dose-dependent inhibition exhibited by the LMWF was observed after each incubation, there was a reverse in the data, which showed up on the cell viability, in LMWF of lower concentrations (<150 µg/mL), and was observed for all repeated independent tests. From 72 h to 96 h, an increased cell viability of approximately 6–10% was observed with the decreasing concentrations of LMWF, indicating that a higher concentration of treatment was required for a better inhibition in the MDA-MB-231 cells. Although not time-dependent, the dose-dependent manner of the inhibitory effect of LMWF was exhibited in all time points. Thus, these data show that the effect of LMWF concentrations on inhibition in the MDA-MB-231 cells, was significant (P_Cn_ = 0.0169), and the effect of time was also very significant (P_Time_ = 0.0046). Due to the reverse of cell viability in LMWF of lower concentration groups, the inhibitory effect of LMWF on the MDA-MB-231 was not considered time-dependent. There was an interaction of the effect of LMWF concentrations and the incubation time (P_Time:Cn_ < 0.0001).

The MCF-7 cells appeared to be much more sensitive to LMWF than the MD-MB-231 cells, with the best inhibitory effect being nearly 20% higher in the MCF-7 group than in the MDA-MB-231 group, and the IC50 value being only 10.54 µg/mL, after 96 h (IC50 was above 300 µg/mL for the MDA-MB-231 cells, by 96 h). The reverse of cell viability, over time, only appeared in the MDA-MB-231cells groups, and not in the MCF-7.

**Figure 7:**
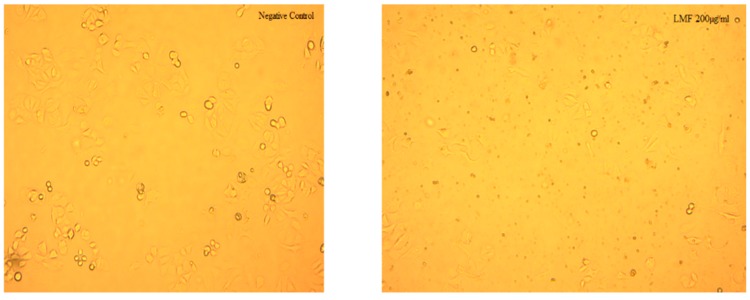
Comparison of the MCF-7 treated with/without 200 µg/mL LMWF, after 48 h.

**Figure 8:**
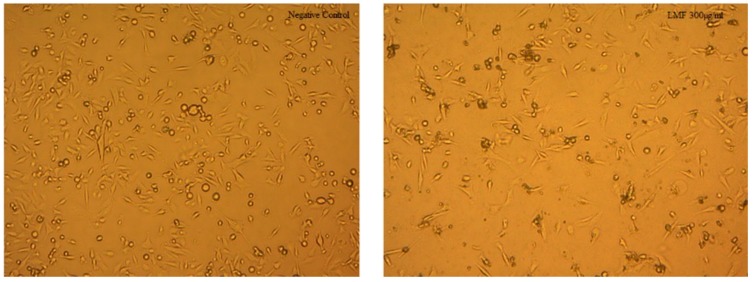
Comparison of the MDA-MB-231 treated with/without 300 µg/mL LMWF, after 72 h.

When observed under microscope, there was a decrease in the population of cells, in both cell lines, after incubation with LMWF (Figure 7 and Figure 8). The decrease was most obvious at LMWF concentrations of 200 µg/mL and 300 µg/mL, respectively.

### 2.5. Cytotoxicity Mechanisms

Mechanism assays were performed on the MDA-MB-231 cell line.

#### 2.5.1. Examination of the LMWF-Induced Apoptosis

**Figure 9:**
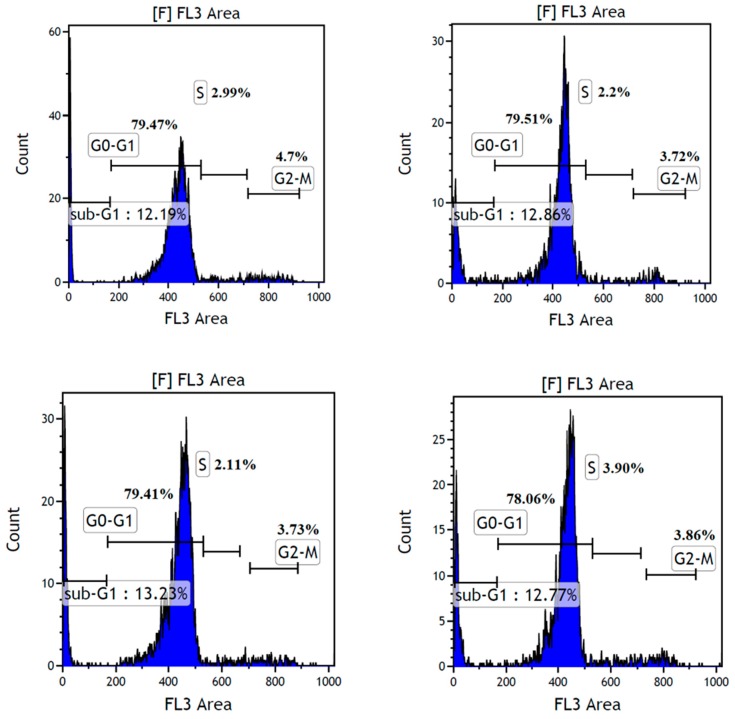
Cell cycle distribution of the HDFa cells treated without LMWF (top left), with 300 (top right), 150 (bottom left and 75 (bottom right) µg/mL LMWF after 72 h.

**Figure 10:**
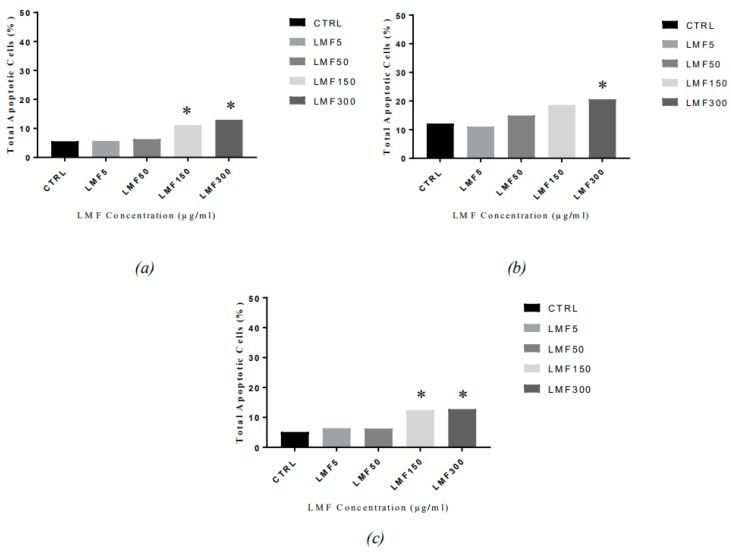
The apoptotic cells distribution in MDA-MB-231 cells after being treated with LMWF (µg/mL) for (**a**) 48 h, (**b**) 72 h, (**c**) 96 h and harvest (* *P* < 0.05 vs negative controls).

HMWF did not show any effect in the viability study, therefore, we only examined the LMWF-induced apoptosis on the MDA-MB-231 cell line. The percentage of the apoptotic cells in the LMWF treated MDA-MB-231 cells and the related hallmarks, were examined in this study. Figure 9 shows no obvious percentage changes (<1%) observed in the G0-G1 phase and the sub-G1 phase, in cells treated with LMWF, compared to the negative controls. Cell cycle arrest was also not observed in the S phase and the G2-M phase, indicating that LMWF is not cytotoxic to the HDFa cells, up to a concentration of 300 µg/mL. As can be seen from Figure 10, apoptosis was significantly induced at LMWF concentrations of 150 µg/mL and 300 µg/mL, at 48 h, 72 h, and 96 h. The increase of the apoptotic cells was most significant in the cells treated with 300 µg/mL LMWF (total apoptotic cells were 13.93% after 48 h, 20.94% after 72 h, and 12.75% after 96 h). The average of this increase was about 8% (8.38%, 8.81%, and 7.6% after 48, 72, and 96 h, respectively) compared to the negative controls, in all three points. Thus, no obvious time-dependent pattern was observed in this assay, while the total apoptotic cell percentage appeared to be correlated to the LMWF in a dose-dependent manner.

**Figure 11:**
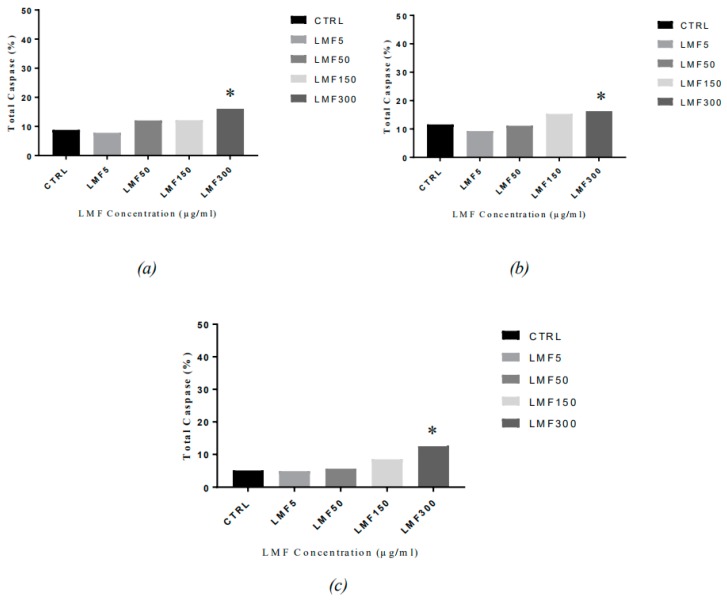
The caspase activation in the MDA-MB-231 cells after being treated with LMWF (µg/mL), for (**a**) 48h, (**b**) 72 h, and (**c**) 96 h, and harvest (* *P* < 0.05 vs negative controls).

Caspase activity has been observed in the fucoidan-mediated apoptosis (Figure 11). From 48 h to 96 h, an increase in the total caspase in the cells has been observed, at each time-point, with an increasing dose of LMWF, indicating that the LMWF-induced cell apoptosis in the MDA-MB-231 is a caspase-dependent apoptosis.

The most obvious increase of the total caspase was also observed in the cells treated with 300 µg/mL LMWF, in all three time points (17.05%, 17.65%, and 12.75% after 48, 72, and 96 h, respectively), and a slightly lower increase in the cells treated with 150 µg/mL LMWF (12.15%, 15.30%, and 8.50% after 48, 72, and 96 h, respectively). Compared to the negative controls (8.80%, 11.6%, and 5.15% after 48, 72, and 96 h, respectively), the increase of total caspase in the cells treated with lower concentrations (50 µg/mL and 5 µg/mL) of LMWF, were generally not obvious. This trend of caspase activation in the cells, corresponds to the trends in the cell apoptosis assay. No obvious time-dependent pattern was observed on the caspase activity, in this assay.

**Figure 12:**
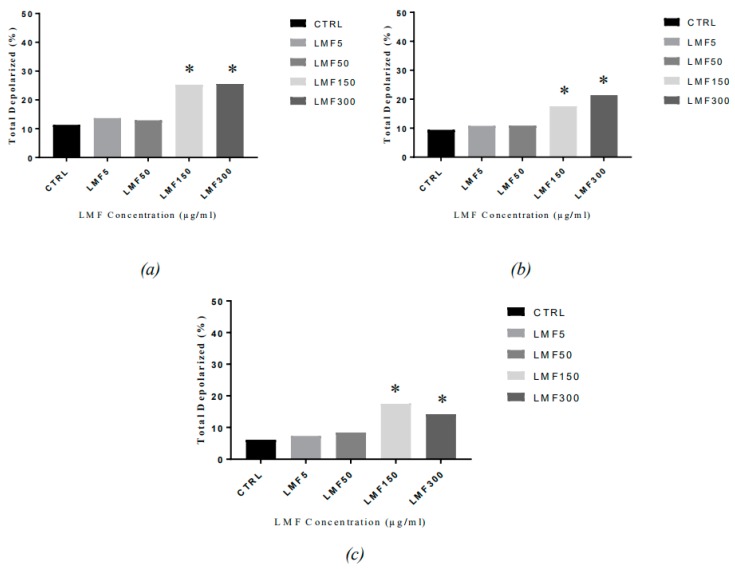
The status of mitochondria in the MDA-MB-231 cells, after being treated with LMWF (µg/mL), for (**a**) 48h, (**b**) 72 h, (**c**) 96 h, and harvest (* *P* < 0.05 vs negative controls).

The loss of mitochondria membrane potential (MMP) is often considered as a sign associated with the early stages of cell apoptosis. In this study, an increase of cells with a depolarised mitochondrial membrane was observed at all three time points, with an increasing dose of LMWF (Figure 12). Compared to the negative controls, the highest concentrations of the LMWF (150 µg/mL and 300 µg/mL, with 26.05% and 25.25%, 22.40% and 18.05%, 14.40% and 17.65%, in the cells, after 48, 72, and 96 h, respectively) significantly doubled the percentage of cells with mitochondria depolarisation, indicating that the MMP had changed during the LMWF-induced cell apoptosis. A minor increase (1–2%) was observed in the percentage of the cells treated with 50 µg/mL and 5 µg/mL LMWF. This finding implied the pathway of the fucoidan-induced apoptosis, which was a mitochondria-mediated apoptosis, therefore, activation of the intrinsic apoptosis pathway was confirmed in the LMWF-induced apoptosis in the MDA-MB-231. No obvious time-dependent pattern was observed on the loss of MMP, in this assay.

#### 2.5.2. Examination of the Nitrosative Stress Parameters in the LMWF-Treated MDA-MB-231 Cells

**Figure 13:**
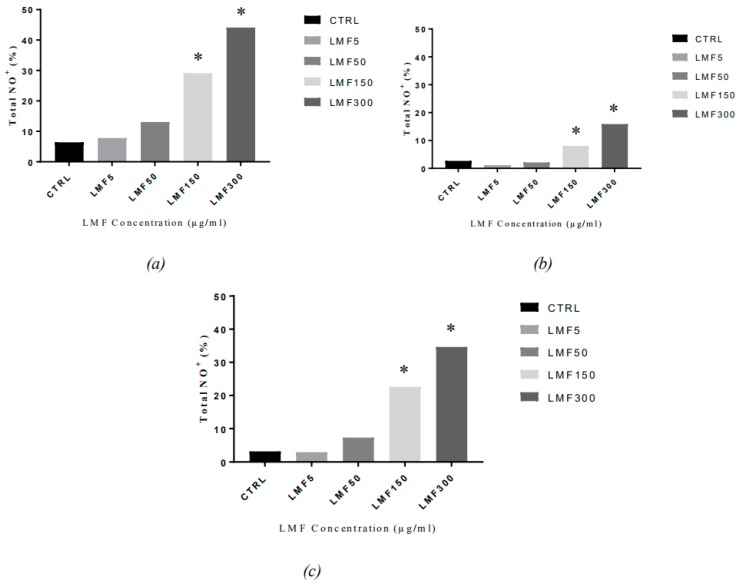
The nitrosative stress parameters in the MDA-MB-231 cells after being treated with LMWF (µg/mL), for (**a**) 48h, (**b**) 72 h, (**c**) 96 h, and harvest (* *P* < 0.05 vs negative controls).

The LMWF seemed to stimulate NO production in the MDA-MB-231 cells (Figure 13). Elevation of nitrosative stress in the cells had been observed, with the treatment of LMWF, at each time-point and appeared to be positively correlated to the dosage of LMWF, which meant it was dose-dependent. The increase of the total nitric oxide positive cells was most remarkable with the 300 µg/mL LWMF, which was roughly seven times higher than that in the negative controls, at 48 and 72 h, and eleven times higher than that in the negative controls at 96 h (the highest level of NO was observed in the cells—at about 37.70%—after an incubation of 48 h, 13.20% after 72 h, and 31.50% after 96 h, compared to the negative controls); at 150 µg/mL, minor increases were observed with a lower LMWF concentration of 50 µg/mL.

### 2.6. Discussion

There are relatively few reports about the toxins and pollutants in fucoidan. Literature suggests that fucoidan and alginate absorb the heavy metals [38]. Hence, the heavy metal content was analysed and compared with the food standard. There is a limited number of reports about the heavy metal and organic pollutants in fucoidan and this study filled this void in the literature. Previous structure-activity-relationship (SAR) studies showed that a higher sulphation contributed to the better anticancer cell activity of fucoidan [39] and that the large MW fucoidan polymer hinders the reaction of the sulphate groups, with cancer cells inside its spherical conformation, as opposed to the loose and linear form of LMWF [19]. Our results indicated that NZ-extracted fucoidan has a good anti-proliferation activity, which was consistent with our recent report where the apparent anti-proliferation results of various cancer cells were obtained [18]. The fucoidans’ anti-cancer activities have been observed against lymphoma, melanoma, colon, liver, lung, and breast cancers [20,40,41]. Furthermore, a recent in vitro and in vivo study, using the NZ-extracted fucoidan also demonstrated that it had synergistic anticancer effect in the melanoma model, when combined with the chemotherapeutic agent lapatinib [42].

In terms of fractions with different molecular weight, many reports suggested that the low molecular weight fraction of the fucoidan had a better bioactivity [43]. A recent clinical trial in cancer patients showed that LMWF, combined with chemotherapeutic agents in the metastatic colorectal cancer, could significantly improve disease control rate [44]. This further strengthened the idea that fucoidan containing a lower molecular fraction should have better bioactivity, which has been seen in the NZ-extracted fucoidan [18,42]. In the present study, a LMWF exhibited a greater suppression on the cell viability, in both the cell lines, whereas, the inhibitory effect of the HMWF had a limited effect on slowing down the growth of cancer cells. Zhang et al. reported a similar trend in the growth inhibition of the MCF-7 and the MDA-MB-231 breast cancer cell lines, while testing the effect of a LMWF derived from the seaweed Mozuku of the Cladosiphon novae-caledoniae Kylin, from the Kingdom of Tonga [25]. Their data showed that the LMWF exerted a 60% growth inhibition in the MCF-7 cells and a 41% growth inhibition in the MDA-MB-231 cells, after the LMWF treatment of 820µg/mL for 96 h, which was similar to the results found in this study. Differences in the highest concentrations of LMWF, in these two different studies, indicated, that the LMWF from the New Zealand *U. pinnatifida* exhibits a better efficacy than the LMWF from Cladosiphon novae-caledoniae Kylin, considering that the LMWF in this study showed a similar inhibitory effect on the same cell lines, using one-third to half of the concentrations, used in Zhang’s treatment (200 µg/mL and 300 µg/mL of LMWF, compared to 820 µg/mL). 

Results of a very recent study demonstrated that low molecular weight mannogalactofucans prepared from a high molecular weight Miyeokgui fucoidan extracted and purified from the dried sporophyll of *U. pinnatifida,* attenuated the growth of the human prostate cancer cells, both in vitro and in vivo [45]. Another very recent study compared the anticancer activity of different molecular weight fucoidan, in many cancer cells, including the MCF- 7 (human breast carcinoma), the AGS (human stomach cancer cell line), and the HepG-2 (Hepatoma cell line), and found that in the case of the MCF- 7 cell line, there was a proliferation inhibition that increased, depending on the concentration of fucoidan. A non-significant difference in the cytotoxicity between the HMWF and LMWF was observed, at a low fucoidan concentration (0.5 mg/mL). However, at a concentration of 2 mg/mL, the cytotoxicity of a high molecular weight fucoidan was 42% and that of a low molecular weight fucoidan was up to 66%. The difference in cytotoxicity became more pronounced when the concentration increased to 4 mg/mL. Similar effects were found in the other investigated cell lines. Moreover, LMWF was shown to have a higher inhibitory effect on the cell transformation [46].

Our results indicated that LMWF is not cytotoxic to HDFa cells, up to a concentration of 300 µg/mL. These results were in accordance with the findings of Hwang et al. which showed that at 5000 μg/mL LMWF exhibited no toxicological indications or mutagenicity [47]. They recommended LMWF to be used as a safe food supplement, as they examined the toxicity of the LMWF extracted from *Laminaria japonica*, in mouse and rat models, by many different methods [47].

On the other hand, in a very recent study, three fucoidan preparations; F (crude fucoidan obtained from *K. crassifolia*), and its fractions F1 and F2, showed very limited efficacy at killing murine hepatocarcinoma Hca-F cells, in vitro. In the in vivo experiment, the crude extract at a dose of 450 mg/kg/day had a significantly restricted lump growth, whereas the fractionated F1 and F2 did not show comparable activities. The researchers speculated that the origin of the anti-neoplastic effect of the crude extract might be contributed by small molecules, such as phenols, that are attached to the native fucoidan [48]. The origin of the fucoidans’ anti-neoplastic effect, as well as the mechanisms, have been investigated and reported by many researchers, such as the suppression of neovascularity, activation of a cell-mediated immunity [6], and the induction of apoptosis [49].

Chemotherapeutic agents have a high potential in inducing apoptosis in various cancer cells, which has become a matter of great interest [50]. Thus, screening for natural products that have the ability to induce apoptosis in cancer cells has now been in progress, to reduce the side-effects and elevate the therapeutic effects in cancer therapy [51]. The present study confirms the LMWF-mediated apoptosis in MDA-MB-231, as a caspase-dependent apoptosis. Zhang et al. hypothesised that the distinct caspase-3 expression may contribute to the different effects of the LMWF on two cell lines [23]. They argued that the MCF-7 cells do not express full-length caspase-3 to induce a positive feedback loop and does not undergo a formation of apoptosomes in vivo, while caspase-3 is expressed in the MDA-MB-231 cells. Overall, the fact that the MCF-7 cells have a higher sensitivity to LMWF than the MDA-MB-231 cells, has been proven in this study. The difference between the p53 statuses of the two cell lines might contribute to their distinction of the LMWF-sensitivity. The p53 in the MCF-7 cell line is the wild type, whereas, in the MDA-MB-231 it is the mutated p53. Tumour cells which harbour mutated p53, are typically more resistant to certain anti-cancer drugs because mutated p53 no longer renders the tumour-suppressing abilities of the wild type p53, which also makes breast cancer, of this type, clinically more challenging to treat [52]. The present study also detected the pathway of the fucoidan-induced apoptosis to be mitochondria-mediated, thus, confirming the LMWF-induced apoptosis to be via the activation of the intrinsic apoptosis pathway. This result is consistent with literature that the intrinsic pathway is the primary pathway in the fucoidan-induced apoptosis in the cancer cell lines [25,53]. 

In the present study, it was confirmed that a LMWF increased the NO production in the MDA-MB-231 cells. A dose-dependent elevation of nitrosative stress in the MDA-MB-231 cells has been observed with the treatment of LMWF in each time-point, and it is positively correlated to the dosage of LMWF. Although the increase in NO was consistent with the dose-dependent inhibitory effect by LMWF on the MDA-MB-231 cells, these cells were functionally unable to carry out the NO-mediated apoptosis [54]. The NO resistance in the MDA-MB-231 cells and the protective role of the low-concentration of NO, in cells [55], may explain why the cell viability of the MDA-MB-231 cells that was inhibited by a LMWF in lower concentrations (<150 µg/mL), was slowly reversed in the methylthiazol-diphenyl-tetrazolium (MTT) assays of this study. This could also provide possible explanation for the relatively lower sensitivity to LMWF in the MDA-MB-231, compared to the MCF-7 cells. To the best of our knowledge, few research projects have studied the fucoidan/LMWF stimulation of NOS in breast cancer cells, while more of the studies focus on the anti-cancer pathway of the fucoidan-activated macrophages and stimulation of iNOS in the macrophages. Considering the fact that iNOS expression was also the main NOS activity in breast cancer, it was suspected that the LMWF may stimulate iNOS in the MDA-MB-231, the same way, as in macrophages. Despite no cytotoxicity of NO to the MDA-MB-231 cells [56], there were some other functions of NO that might have a link with the previously-reported anti-cancer properties of the fucoidan on the MDA-MB-231 cells, including metastasis inefficiency [57] and the synergy with cytotoxic chemotherapeutic agents [57,58]. Therefore, further mechanism studies are suggested to better examine the relationship between an increased NO in the breast cancer cells and the fucoidan anti-cancer properties.

These findings reinforce the necessity of studying ways aiming to retain and improve the fucoidan functional properties, such as an increasing LMWF percentage and degree of sulphation, in the different industrial processes [59].

## 3. Materials and Methods

### 3.1. Materials

Five commercially-available fucoidan products extracted from the *U. pinnatifida* were compared with the *U. pinnatifida* fucoidan (S) (≥95%, CAS: 9072-19-9, from North America) purchased from Sigma Aldrich (St Louise, MO, USA): (1) Crude fucoidan from our pilot-scale production in New Zealand (S1); (2) fucoidan (with a claimed purity of 75.5%) purchased through the Alibaba website from a Chinese manufacturer (Anhui Minmetals Development I/E Co., Ltd., Hefei, China) (S2); (3) fucoidan produced by a local New Zealand business, Matakana SuperFoods (S3), who sources *U. pinnatifida* from Patagonia; (4) fucoidan (GF100, minimal purity 75%) (S4) purchased from Glycomix UK, a distributor for Marinova, the leading commercial producer of fucoidan in Australia; and (5) fucoidan purchased from Leili Ltd. (Beijing, China) (S5). Cell mechanism assays kits were purchased from Merck Millipore.

### 3.2. Extraction of the Crude Fucoidan from Local Pilot-Scale Production

The method was established based on the existing literature [60,61] and up-scaled to an industrial scale [18]. In brief, seaweed was minced and put into water maintained at 70 °C, overnight. The resulting liquid mixture was centrifuged to remove solids. Calcium chloride was added to eliminate alginate. The remaining supernatant was added to the excessive amount of ethanol to precipitate the fucoidan out of the solution. Then, the fucoidan was collected and dried into the powder form.

### 3.3. Determination of the Chemical Composition

The phenol-sulfuric acid method was used for the quantification of the total carbohydrate content in the fucoidan samples, using fucose as a standard [62,63]. The BaCl_2_-gelatin method was used to determine the sulphate content, using K_2_SO_4_ as the standard, after treating the samples with 1M HCl at 105 °C, for 12 h, for sample hydrolysis [64]. The carbazole-sulfuric acid method was used for measuring the uronic acid, using glucuronic acid as the standard [65], and a Bicinchoninic acid protein assay kit (Beyotime Biotech., Jiangsu, China) was used to estimate the protein content. The Association of Official Agricultural Chemists (AOAC) official method was used to estimate the content of the crude fat [66] and the Chloroform–methanol (3:1, *v*/*v*) method was used to estimate the ash content [67].

### 3.4. Determination of the Monosaccharide Composition

High-performance anion exchange chromatography (HPAEC), associated with a pulsed amperometric detector (HPAEC-PAD) (Thermo Fisher Scientific, Waltham, MA, USA), were used to separate and quantify the monosaccharide constituents of the polysaccharides [68]. Fucoidan (5 mg) was hydrolysed in sulfuric acid (12 M, 0.5 mL), for half an hour, in an ice bath. To achieve a concentration of 2 M sulfuric acid, distilled water was used to dilute the mixture, which was then placed in an oil bath, at 100 °C, for 2 h. Prior to injection, the hydrolysate was diluted four-fold, before filtering through a 0.22 μm syringe tip filter (SHIMADZU-GL, Wonda Disc II, MCE; Shimadzu, Kyoto, Japan).

Dionex ICS-2500 system (Dionex Corp., Sunnyvale, CA, USA) was used to perform the HPAEC-PAD analysis, the system was equipped with CarboPac™ PA20 analytical column (250 mm × 4 mm I.D., Dionex Corp., Sunnyvale, CA, USA) and the CarboPac™ PA20 guard column (50 mm × 4 mm I.D., Dionex Corp., Sunnyvale, CA, USA). For the mobile phase, CH_3_COONa (1 M), H_2_O, and NaOH (250 mM) were used. The elution program comprised the following steps, at a flow rate of 0.5 mL/min: 0 to 20 min (0.8% NaOH and 99.2% H_2_O), 20 to 20.1 min (0.8% NaOH, 94.2% H_2_O, and 5% CH_3_COONa), 20.1 to 30 min (0.8% NaOH, 74.2% H_2_O, and 20% CH_3_COONa), and 30 to 50 min (80% NaOH and 20% H_2_O). The column was kept at 30 °C. 

Sugar standards included were ribose, D-(-)-arabinose, L-rhamnose, L-(-)-fucose, D-(+)-glucose, D-fructose, D-mannose, D-galactose, D-(+)-xylose, D-glucuronic acid, and D-(+)-galacturonic acid (Merck Co., Darmstadt, Germany; Sigma Chemical, St. Louis, MO, USA).

### 3.5. Molecular Weight Distribution

Determination of the molecular weight distribution of the fucoidan was performed by high performance gel permeation chromatography (HPGPC). An Agilent 1260 HPLC system (Agilent, Santa Clara, CA, USA) was used, including a Refractive Index Detector (RID, DEAA602884), a Variable Wavelength Detector (VWD, DEABB05037), and an Ultrahydrogel^TM^ Linear column (7.8 mm × 300 mm) (Waters, Milford, MA, USA). The column was maintained at 35 ± 0.1 °C. The mobile phase was NaNO_3_/0.02% NaN_3_ (0.1 M), and the flow rate was 0.6 mL/min. The polysaccharides molecular weight distribution was calculated from the calibration curve provided by a series of dextran standards (T-10, T-40, T-70, T-500 and T-2000). They were bought from Pharmacia Biotech (Uppsala, Sweden). All other solvents and chemicals used were of the analytical reagent grade and were bought from Sinopharm Chemical Reagent Co., Ltd. (Shanghai, China). The wavelength was 280 nm for both RID and VWD.

### 3.6. Elemental and Organic Pollutants Analysis

The contents of K, Ca, Na, Mg, and S were estimated using inductively-coupled argon plasma atomic emission spectroscopy (ICP-AES) (Optima 5300DV, PerkinElmer, Waltham, MA, USA), and the contents of Hg, Pb, Cr, Cd, and As were estimated through atomic fluorescence spectrometry (AFS-230E, Shanghai, China). The polysaccharides were digested in HNO_3_/HClO_4_ (4:1, *w/w*) [69] and filtered through a 0.45 μm membrane, prior to detection. The fluoride content was determined, based on the GB/T 5009.18-2003 [70]. The content of chloride and polychlorinated biphenyls (PCBs) were analyzed, based on the GB/T 5009.190 (China National Food Standard – Seafood). In the case of S4 (Marinova fucoidan), some heavy metal testing was done by a third-party laboratory (AsureQuality Ltd., Auckland, New Zealand), using inductively-coupled argon plasma mass spectroscopy (ICP-MS).

### 3.7. Preparation of Fucoidan Fractions and Stock Solution

Different molecular weight fucoidan fractions were obtained through dialysis. To make 1% solution, fucoidan was dissolved in water. The solution was kept at 4 °C, for three days, in a 10,000 Dalton dialysis bag. The solution was collected out of the bag and frozen dried to obtain <10,000 Da LMWF. The solution was put into a 300 kDa dialysis bag and kept at 4 °C, for another three days, inside the bag. Collection of the solution out of the bag and freezing it dry was performed to obtain the 10–300 kDa fraction. The solution in the bag was collected and frozen-dried to have the HMWF. The LMWF was dissolved in the cell culture RPMI 1640 base medium (with 1% Penicillin-Streptomycin, 1% L-glutamine and 10% fetal bovine serum) and the HMWF was dissolved in medium 106 (with 1.96% LSGS). The HMWF was dissolved to a final concentration of 300 μg/mL, as a stock solution. The LMWF was dissolved to a final concentration of 600 μg/mL, as a stock solution. Fucoidan stock solutions aliquots were distributed in micro-tubes and were stored at −80 °C, wrapped up in aluminium foil. 

### 3.8. Cell Lines

Estrogen receptor (ER)-positive MCF-7 and ER-negative MDA-MB-231 cells were used in this study. HDFa is a normal cell line used to examine toxicity of fucoidan.

All cell lines used in the study were stored in liquid nitrogen or at −80 °C. After thawing, the cell lines were kept in 37 °C incubator, with 5% CO_2_ humidified air, maintained in 25 or 75 cm^2^ tissue culture flasks, which contained the completed growth culture medium (5 mL or 12 mL).

### 3.9. Cell Proliferation Assay

MTT assay was used to determine cell viability, the test was based on the mitochondrial dehydrogenases reduction of the tetrazolium salt to a blue-coloured formazan, in the viable cells [71]. Cells were seeded at densities of 5000 cells/well, for 18 h, in 96-well plates. Then the incubation of cells with 100 μL of each concentration of treatments (HMWF or LMWF), was performed. After incubation for 48, 72, and 96 h, 10 μL of the MTT stock solution was added to each well in the plates, before placing them back into the 37 °C incubator, for 4 h. After incubation, a purple precipitate was clearly visible under an inverted microscope. The supernatant was aspirated and replaced with 100 μL/well of the DMSO for the formazan salt to be dissolved. Incubation at 37 °C for 20 to 30 min was performed and the colour intensity of the formazan solution was estimated, using a microplate reader (FLUOstar Omega, Alphatech Systems Ltd., Auckland, New Zealand), at 540 nm.

### 3.10. Cell Cycle Assay

Cells were seeded into six-well plates at a density of 50,000 cells/mL, with 2 mL cell suspensions contained in each well. A parallel group (plate) was set in the experiment. The two plates were kept in a 37 °C incubator, for about 18 h. The treatment was prepared using the 600 μg/mL LMWF stock (of medium 106) and a complete culture medium 106, for the HDFa, diluted into the concentrations of 300 μg/mL, 150 μg/mL, and 75 μg/mL. the old medium was removed from the plates, prior to adding 2 mL LMWF of each concentration, into the corresponding wells. Two millilitres of fresh complete medium was added instead of the LMWF, in the control wells. Medium/treatment in each well was transferred into a 15 mL labelled centrifuge tubes. Five hundred microliters of PBS were added and then collected into the relevant tubes. After wash, 500 μL TrypLE™ Express Enzyme were added and the plate was placed back in the incubator, for 5 min. One millilitre medium was added into each well and the mixture was transferred into the corresponding tubes. Another 500 μL PBS were added and each well was washed again and then collected. Then centrifugation of all cells containing the tubes, at 1200 RPM for 5 min, at 4 °C, was performed. The supernatant was discarded, another centrifugation of 5 min was performed, before adding 1 mL PBS into each tube. Most of the supernatant was discarded in each tube. One millilitre ice-cold 80% ethanol was added, then, for 72 h both plates were kept incubated with the treatment, before they were harvested and stored at −20 °C, for the following analysis. The cells were washed twice each time with 3 mL ice-cold PBS, after discarding the ethanol. In the process of the propidium iodide (PI) staining, the supernatant was discarded from each well and 1 mL permeabilizing solution was added to each tube. All cells in the tubes were transferred to the test tubes and kept incubated for 30–45 min, at 37 °C. Five microgram per millilitre of PI (5 μL to 1ml in each tube) was added to each test tube, after permeabilisation, and kept for 5 min. All samples were tested using the flow cytometer.

### 3.11. Cell Mechanism Assays

#### 3.11.1. Cell Samples Preparation

The MDA-MB-231 cells were seeded at a density of 45,000 cells/mL in 6-well plates, with 2ml cell suspension in each well. For about 18 h, all plates were kept in a 37 °C incubator. The treatment was diluted into the final concentrations of 300 μg/mL, 150 μg/mL, 50 μg/mL, and 5 μg/mL and kept in 15 mL centrifuge tubes. 2 mL LMWF solution of each concentration was added into the corresponding wells. Two millilitres of fresh complete medium was added into the wells of the negative and the positive control (for the multicaspase assay). Plates were kept incubated with treatment for 48, 72, and 96 h respectively, with one plate for one time-point. Half an hour before harvesting the cells, the old medium in the positive control well was removed and changed into 2 mL 10 mM HP medium solution. Cells were incubated for 30 min, then the old medium/treatment was removed, followed by addition of 1 mL of PBS, into each well. After a gentle rinse, the PBS in each well was collected into the corresponding, labelled 15 mL centrifuge tubes, then, 0.5 mL TrypLE™ Express Enzyme were added into each well. During detaching, the plate was placed back in the incubator, for no more than 5 min, until most cells were detached from the wall/bottom. One millilitre complete cell culture medium was added to terminate the detachment, and the mixture in each well was collected. Another 1 mL PBS was added to each well, rinsed, and transferred into the tubes to ensure all cells were collected. Tubes were centrifuged at 1200 RPM, at room temperature, for 5 min, and the supernatant was discarded. To resuspend the cells, a complete culture medium was added into each tube. Cells were counted for cell suspension, in each tube, using the haemocytometer, and the cell concentrations of each sample were adjusted to be in the range of 1 × 105–5 × 105 cells/mL (by dilution or centrifugation).

#### 3.11.2. Cell Viability Assay

The viability of the harvest cells was measured. The cell concentrations in each sample were rechecked and adjusted to 1 × 105–5 × 105 cells/mL, for the following assays. The Muse™ Count and Viability Reagent was stored at 4 °C. Depending on the concentration of the cell sample, proper amount of this reagent was added into each microcentrifuge tube. Cell suspension in each sample was remixed. The required amount of cell suspension was then removed into the test tubes and labelled. All tubes were kept incubated for 5 min, at the room temperature, to ensure a complete staining. Fifty microlitres of cell suspension were added into the 450 μL reagent, in each test tube, and incubated for 5 min. The mixture in each test tube was remixed, then loaded onto the MuseTM Cell Analyser for measurement.

#### 3.11.3. Cell Apoptosis Assay

To study the fucoidan-induced cell apoptosis in the MDA-MB-231cell line, three assays were applied, namely Annexin V and Dead Cell Assay, the MultiCaspase Assay, and the MitoPotential Assay.

##### Annexin V and Dead Cell Assay

The Muse™ Annexin V and Dead Cell Reagent was stored at 4 °C and pre-warmed to room temperature, before use. Hundred microlitres of this reagent were added into each test tube. The cell suspension in each sample was remixed by pipetting 3–5 times. Hundred microlitres of cell suspension were then removed into the reagent, in the test tubes, and was labelled. All tubes were kept incubated in the dark for 20 min (at room temperature), to ensure a complete staining. The mixture in each test tube was gently remixed again, then loaded onto MuseTM Cell Analyser for measurement.

##### Multi-Caspase Assay

Five microlitres of the Muse™ multi-caspase reagent working solution were added to each labelled tube. Tubes were then loosely caped and incubated in the 37 °C incubator, for 30 min, with 5% CO_2_. After incubation, 150 μL of Muse™ caspase 7-AAD working solution were added to each tube. Samples were incubated at room temperature for another 5 min, protected from light. Samples were remixed thoroughly and then run on the MuseTM cell analyser.

##### MitoPotential Assay

Ninety-five microlitres of mitopotential working solution were added to each labelled tube. Cells were incubated for 20 min, in a 37 °C CO_2_ incubator. Five microlitres of the Muse MitoPotential 7-AAD reagent were added to each well and mixed. Samples were incubated for another 5 min, at room temperature. Samples were remixed thoroughly and run on the MuseTM cell analyser.

#### 3.11.4. Nitrosative Stress Measurement

The levels of nitric oxide were evaluated using the MuseTM nitric oxide Kit. Ten microlitres of the prepared cell suspension were added into each test tube and labelled. Hundred microlitres of the Nitric Oxide reagent working solution were added to each tube. Samples were incubated for 30 min, in the 37 °C incubator, with 5% CO_2_. Ninety microlitres of the 7-AAD working solution were added into each tube after incubation. Samples were incubated for another 5 min at room temperature, protected from light. Samples were remixed thoroughly and run on the MuseTM Cell Analyser.

### 3.12. Statistical Analysis

Statistical analyses was performed using the GraphPad Prism (GraphPad Software, San Diego, CA, USA) software package. Results were expressed as mean ± SEM (sample n = 3 with triplicate analysis done on each sample). One-way ANOVA was used to perform the analyses of variance, with post hoc Tuckey’s test for significant differences. A nominal two-sided *P* < 0.05 was used to assess significance.

MTT assays were performed more than three times. Statistical differences in multiple groups were determined by one-way ANOVA (Analysis of Variance) and Linear Mixed Effects Model (LMEM) with the R-Studio^®^ software (version 3.5.0). Differences of *P* < 0.05 were considered significant and *p* < 0.01 were considered to be very significant.

The software applied in cell cycle results analysis was Kaluza^®^ Flow Cytometry Analysis Software (Version 1.3) purchased from the Beckman Coulter.

## 4. Conclusions

Our study clarified the differences regarding the proximate composition, molecular weight distribution, as well as the contents of the elements and the organic pollutants, among the six fucoidans. Results showed that each fucoidan has its unique molecular composition, hence, each may possess a different bioactivity. One of the most obstructive factors for determining the fucoidans’ bioactivities is the molecular weight. The LMW fraction detected in the New Zealand crude fucoidan (S1) was larger than that of S, and this fraction was unique, compared to the other studied fucoidans. We demonstrated that the New Zealand LMWF had a better bioactivity than the HMWF, in the two breast cancer cell lines, without any toxicity on the normal fibroblasts. The significantly different cytotoxic effects of the HMWF and LMWF in, both, the MCF-7 and MDA-MB-231 cell lines may be attributed to a large molecular size HMWF, which might hinder its entry through the cancer cell membrane. The LMWF induced caspase-dependent apoptosis in the MDA-MB-231 cells, and it was activated through the mitochondria-mediated (intrinsic) pathway. The apoptosis was significantly induced by higher doses of LMWF, while LMWF of low doses presented only minor effects in apoptosis and apoptosis-related activities, which was consistent with the MTT results. Both, the non-toxic properties of LMWF and the potential of the LMWF-stimulated NO production, in the tumour cells, on the sensitisation of tumour cells to chemotherapeutic agents, were promising qualities for LMWF to become a candidate, in combination with chemotherapy, for treating breast cancer. Taken all together, the LMWF from the New Zealand *U. pinnatifida* had the potential to be considered a promising nutraceutical that could be used as a supplement for cancer.

## 5. Future Research

The anti-cancer potential of the LMWF from New Zealand *U. pinnatifida* has been confirmed in this study, and the inhibitory effects on the two breast cancer cell lines, were considerable but not enough to treat breast cancer alone. A combined study for the LMWF and other breast cancer therapeutic agents is suggested to test the synergy effects. More data are required to fill in the knowledge gap of this field, as a combined therapy is still critical, to allow better suppression of tumour growth and to reduce the risk of drug resistance. Additionally, the determination of whether the LMWF-induced NO increase, contributes to the anti-cancer properties of fucoidan on the MDA-MB-231 cells, may warrant further mechanism studies in areas such as, anti-metastasis and synergy effects.

## Figures and Tables

**Table 1:** Chemical composition of the six fucoidan products: S = Undaria pinnatifida fucoidan (≥95%, CAS: 9072-19-9) purchased from Sigma Aldrich; S1 = crude fucoidan from our pilot industrial-scale production in New Zealand; S2 = fucoidan purchased through the Alibaba website from a Chinese manufacturer; S3 = fucoidan produced by a local New Zealand business, Matakana SuperFoods; S4 = fucoidan (GF100, minimal purity 75%) produced by Marinova Ltd. (Cambridge, Tasmania, Australia); S5 = fucoidan purchased from Leili Ltd. (Beijing, China); all percentages are on a dry basis.

Sample	Total Sugar (%)	Uronic Acid (%)	Protein (%)	Sulphate (%)	Ash (%)	Crude Fat (%)	Monosaccharide Composition			
							Fuc (%)	Gal (%)	Glc (%)	GlcA (%)
S	96.83 ± 1.71	1.61 ± 0.06	tr	25.59 ± 0.63	16.61 ± 2.13	nd	27.44 ± 0.45	25.34 ± 0.70	nd	nd
S1	88.16 ± 1.85	1.76 ± 0.02	1.28 ± 0.02	19.68 ± 1.60	32.96 ± 0.04	1.60 ± 0.06	19.50 ± 0.35	21.20 ± 0.67	nd	tr
S2	89.94 ± 0.32	3.80 ± 0.37	0.25 ± 0.10	nd	4.93 ± 0.03	0.62 ± 0.09	nd	nd	96.71 ± 0.74	nd
S3	44.67 ± 0.15	2.39 ± 0.01	2.09 ± 0.05	15.48 ± 0.50	25.42 ± 0.05	0.29 ± 0.11	13.83 ± 0.51	13.24 ± 0.45	tr	2.46 ± 0.10
S4	80.00 ± 1.39	2.65 ± 0.03	2.41 ± 0.10	20.96 ± 0.85	19.49 ^c^	0.89 ± 0.27	20.35 ± 2.62	19.26 ± 2.79	tr	2.02 ± 0.11
S5	78.75 ± 0.70	4.15 ± 0.12	3.08 ± 0.0058	20.60 ± 0.23	24.33 ± 0.23	2.3 ^c^	19.23 ± 1.26	21.00 ± 0.20	6.38	nd

nd = not detected; tr = trace; ^c^ not determined in parallel due to insufficient sample.

**Table 2:** Chemical composition of each isolated fraction.

Fraction	Total Sugar (%)	Uronic Acid (%)	Protein (%)	Sulphate (%)	Ash (%)
F_<10k_	2.76 ± 0.31	1.42 ± 0.03	7.04 ± 0.47	16.62 ± 1.31	74.39 ± 0.33
F_10k–300k_	11.20 ± 0.39	2.44 ± 0.05	9.57 ± 0.43	7.17 ± 1.96	53.61 ± 0.35
F_>300k_	53.14 ± 0.13	2.53 ± 0.08	7.53 ± 1.03	30.75 ± 1.83	19.47 ± 0.71

**Table 3:** Content of elements and organic pollutants (mg/kg). S = Undaria pinnatifida fucoidan (≥95%, CAS: 9072-19-9) purchased from Sigma Aldrich; S1 = crude fucoidan from our pilot industrial-scale production in New Zealand; S2 = fucoidan purchased through the Alibaba website from a Chinese manufacturer; S3 = fucoidan produced by a local New Zealand business, Matakana SuperFoods; S4 = fucoidan (GF100, minimal purity 75%) produced by Marinova Ltd. (Cambridge, Tasmania, Australia); S5 = fucoidan purchased from Leili Ltd. (Beijing, China).

Elements and Pollutants	Sigma Sample (S)	S1	S2	S3	S4	S5
S	102,750	83,700	4200	60,400	86,600	76,650
K	2700	53,700	14,800	61,300	29,900	26,400
Ca	1100	46,400	310	4240	13,500	44,100
Na	80,200	10,800	6800	27,230	24,700	2200
Mg	1900	1200	6800	4500	7100	2900
Cr	tr	1.0	0.28	0.51	0.71 ^b^	7.4
Pb	tr	4.3	15	6.9	0.11 ^b^	9.2
Cd	tr	0.50	<0.10	1.1	1.7 ^b^	0.84
Hg	tr	0.076	0.29	0.11	0.14 ^b^	0.56
As	tr	1.8	1.3	7.3	5.5 ^b^	0.76
Cl	- ^a^	27,800	23,800	33,500	- ^a^	210
Fluoride	- ^a^	226,600	92,000	59,000	- ^a^	393,900
PCB	- ^a^	nd	nd	nd	- ^a^	nd

^a^ not determined due to insufficient sample; ^b^ sample tested by a third party laboratory (AsureQuality Ltd., Auckland, New Zealand); nd = not detected.
